# Genomic variants exclusively identified in children with birth defects and concurrent malignant tumors predispose to cancer development

**DOI:** 10.1186/s12943-023-01828-5

**Published:** 2023-08-05

**Authors:** Yichuan Liu, Hui-Qi Qu, Xiao Chang, Frank D Mentch, Haijun Qiu, Xiang Wang, Amir Hossein Saeidian, Deborah Watson, Joseph Glessner, Hakon Hakonarson

**Affiliations:** 1 Center for Applied Genomics (CAG), Children’s Hospital of Philadelphia, 3615 Civic Center Blvd Abramson Building, Philadelphia, PA 19104 USA; 2 Department of Pediatrics, The Perelman School of Medicine, University of Pennsylvania, Philadelphia, PA 19104 USA; 3 Division of Human Genetics, Children’s Hospital of Philadelphia, Philadelphia, PA 19104 USA; 4 Division of Pulmonary Medicine, Children’s Hospital of Philadelphia, Philadelphia, PA 19104 USA; 5 Faculty of Medicine, University of Iceland, Reykjavik, Iceland

**Keywords:** Pediatric cancer development, Birth defects, Sequencing

## Abstract

Children with birth defects (BD) express distinct clinical features that often have various medical consequences, one of which is predisposition to the development of cancers. Identification of the underlying genetic mechanisms related to the development of cancer in BD patients would allow for preventive measures. We performed a whole genome sequencing (WGS) study on blood-derived DNA samples from 1566 individuals without chromosomal anomalies, including 454 BD probands with at least one type of malignant tumors, 767 cancer-free BD probands, and 345 healthy individuals. Exclusive recurrent variants were identified in BD-cancer and BD-only patients and mapped to their corresponding genomic regions. We observed statistically significant overlaps for protein-coding/ncRNA with exclusive variants in exons, introns, ncRNAs, and 3’UTR regions. Exclusive exonic variants, especially synonymous variants, tend to occur in prior exons locus in BD-cancer children. Intronic variants close to splicing site (< 500 bp from exon) have little overlaps in BD-cancer and BD-only patients. Exonic variants in non-coding RNA (ncRNA) tend to occur in different ncRNAs exons regardless of the overlaps. Notably, genes with 5’ UTR variants are almost mutually exclusive between the two phenotypes. In conclusion, we conducted the first genomic study to explore the impact of recurrent variants exclusive to the two distinguished clinical phenotypes under study, BD with or without cancer, demonstrating enrichment of selective protein-coding/ncRNAs differentially expressed between these two phenotypes, suggesting that selective genetic factors may underlie the molecular processes of pediatric cancer development in BD children.

**Supplementary Information:**

The online version contains supplementary material available at 10.1186/s12943-023-01828-5.

Pediatric cancer is relatively rare, however, children diagnosed with malignant tumors present a severe clinical situation, which takes a significant toll on affected individuals, families, health care system, and society world-widely. It is well known that children with birth defects (BD) are more likely to develop cancers either during childhood or later in life. Large population-based studies show that the risk is about 2.5-fold higher in BD children compared to children without BD independent of underlying chromosome anomalies [1]. More recent statistical data from the Centers for Disease Control and Prevention (CDC) show that the BD rate is approximately 3% in United States (https://www.cdc.gov/ncbddd/birthdefects/index.html), therefore the cancer risk in BD children without chromosomal anomaly cannot be neglected considering the significant effect size.

In a previous study we reported on genetic variants associated with pediatric cancer in BD children [2]. Whether these variant/biomarkers are predictive of cancer incidence in BD children remains unknown. Moreover, whether these variants impact their corresponding protein-coding/non-coding RNAs and/or further alter underlying biological pathways of malignant tumor development in children with BD remains unknown. In addition, it remains to be determined whether the targets of these exclusive recurrent variants (ERVar), such as the corresponding protein-coding/non-coding RNAs, are exclusively seen in children with BD-cancer compared to those with BD-only: (1) If so, it is important to know if there is any specific functional pathways involved; (2) If not, are there any differences among the overlapped genes, or do the shared genes lead to totally different clinical phenotypes? To address these issues, we assembled one of the largest pediatric oncology and birth defect cohort of children, as a part of the Gabriella Miller Kids First program project (https://kidsfirstdrc.org/), to identify ERVars for two clinical phenotypes (BD-cancer vs. BD-only), respectively, and explore the impact from related protein-coding/non-coding RNAs and their underlying pathways. We identified ERVars by whole genome sequencing (WGS) of 1221 probands without chromosomal anomalies, including 454 BD patients with at least one type of malignant tumors (BD-cancer) and 767 BD patients without any known cancers (BD-only), with additional 345 healthy individuals who are parents/siblings of the probands as the controls. The two BD groups were selected from the same cohort of patient recruitments, and ethnic backgrounds showed no statistical differences. Cases officially diagnosed with carrying cancer driver mutations were excluded in this study. The results of all coding ERVars were provided in Supplementary Table 1 for BD-cancer and BD-only patients, respectively.

## Significant overlap for targets gene/non-coding RNAs with ERVars

To better understand the mechanisms of malignant tumor developments at the molecular level, we searched for variants that are associated with cancer risk such as ERVars in pediatric BD patients with malignant tumors without chromosome abnormalities. When comparing BD that are typically identified at birth to pediatric cancers that often develop later in life, it is important to note that not all children with BD will develop malignant tumors, although they may have a higher risk compared to the general population. Certain genes associated with BD or underlying molecular mechanisms may contribute significantly to tumor growth. The rationales are that recurrent variants effectively eliminate false positives, and variants that are exclusively seen in the two medical conditions (BD-cancer vs. BD-only) would inform the disease pathogenesis. Typically, the ERVars were mapped to mutually exclusive protein-coding/non-coding RNAsfor two distinguished clinical phenotypes (BD-cancer vs. BD-only), as causative ERVars may lead to the activation of different pathways related to tumor development/progression [3]. A major finding of this study is that for genes and genetic variants that overlapped between the two phenotypes, we observed statistically significant overlaps for ERVars located in exons, introns, ncRNAs and 3’UTR regions (P = 6.3E-20 for genes with exonic ERVars, P = 1.1E-57 for intronic ERVars, P = 4.2E-82 for ncRNA ERVars, and P = 1.3E-14 for 3’UTR ERVars) (Supplementary Fig. 1). Therefore, the development and progression of malignant tumors in BD children are likely due to compounded effects from, i.e., different protein-coding/non-coding RNAs with ERVars, plus pleiotropic functional effects of protein-coding/non-coding RNAs. Another notable finding is that genes with 5’UTR variants are almost mutually exclusive for the two clinical phenotypes, i.e., only 4 genes (*SCN3A, MDGA1, GDI2, FAM20A*) overlapped, out of 200 and 290 genes in the two phenotypes respectively.

## Effects of coding ERVars in overlapped genes

**Fig. 1:**
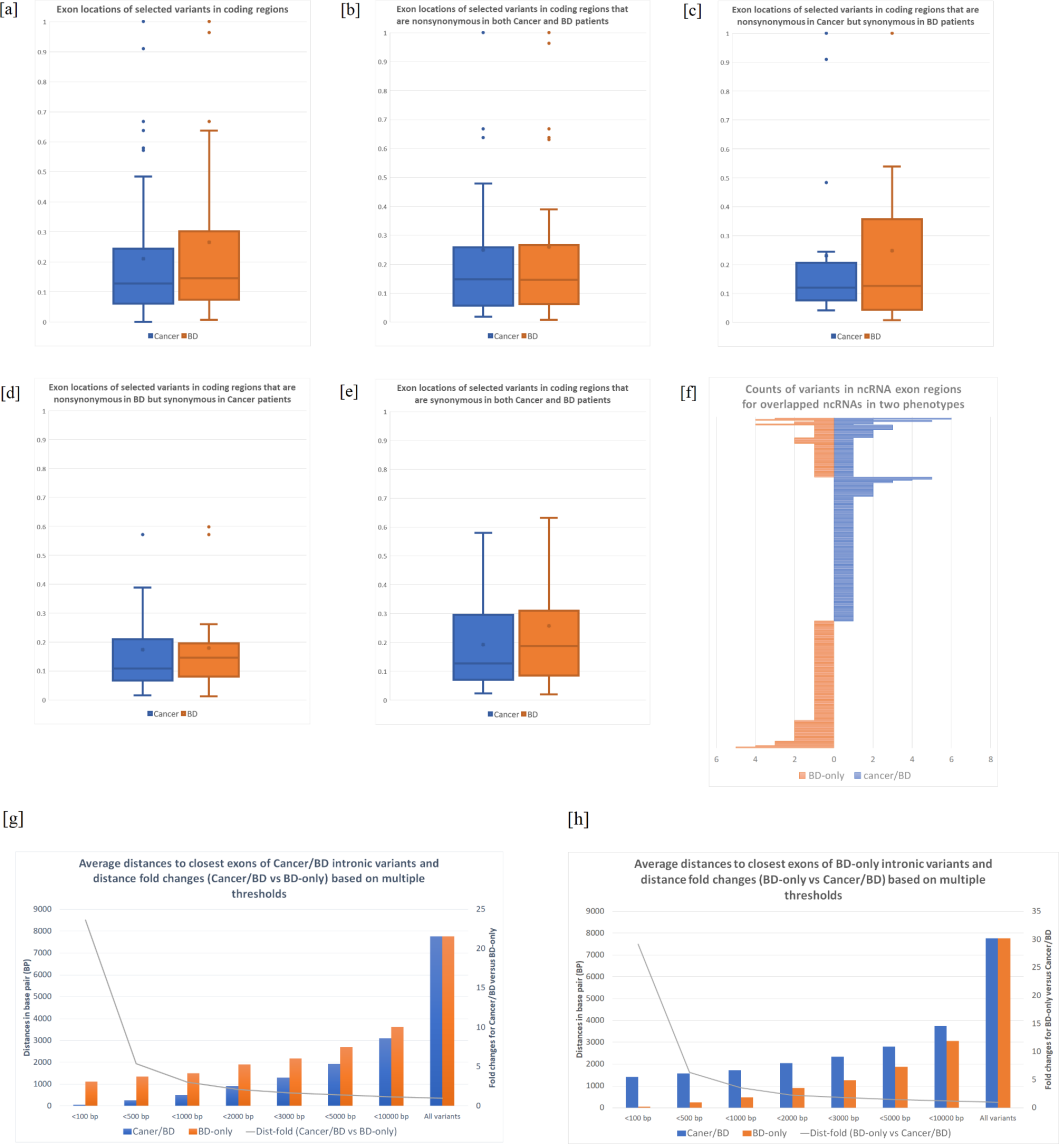
Comparisons of ERVars between BD-cancer and BD-only patients. [**a**] all normalized exonic ERVars; [**b**] nonsynonymous variants in BD-cancer and BD-only; [**c**] nonsynonymous variants in BD-cancer but synonymous in BD-only; [**d**] synonymous variants in BD-cancer but nonsynonymous in BD-only; [**e**] nonsynonymous variants in BD-cancer and BD-only; [**f**] counts for ncRNA exonic ERVars in BD-cancer versus BD-only patients; [**g**] average distances (bp) in multiple thresholds when distances sorted in ascending orders for BD-cancer intronic variants; [**h**] average distances (bp) in multiple thresholds when distances sorted in ascending orders for BD-only intronic variants

From 95 overlapped genes with exonic ERVars, the variants in BD-cancer patients are mapped more frequently to the earlier exons compared to the BD-only group after frequency normalization (Fig. 1A). As suggested, exonic variants in BD-cancer patients tend to occur in the earlier part of mRNA transcripts. We further stratified variants into two groups: exonic variants causing change of protein sequence (missense, frameshifts, stop codon gain/loss) vs. those without amino acid alternation (synonymous). Interestingly, nonsynonymous variants in the two phenotypes showed no difference in exonic locations (Fig. 1B). In contrast, a tendency of different pattern was observed in synonymous variants in BD-cancer patients (Fig. 1C and E). Synonymous variants have been taken as functionally neutral as causing no change of the protein sequence. Nonetheless, recent studies suggest their significant effects involving RNA splicing, stability, folding, translation, or co-translational protein folding in pediatric cancer [4]. An example is the gene *UGT1A8* involved in endometrial cancer [5], which has been identified as a potential risk factor for colorectal cancer [6]. This study showed that the synonymous variants occur in the first exon of *UGT1A8* in BD-cancer patients and the last exon in BD-only patients, implying that synonymous variants may have more functional effects in malignant tumor development in BD children. To further validate our findings, we conducted an additional analysis using a gene set consisting of 565 well-known pediatric cancer driver genes [7]. Through this analysis, and we replicated a significant number of pediatric cancer driver genes, with 37 genes containing coding ERVars (Supplementary Table 2).

## Effects of non-coding ERVars in overlapped genes

ERVars in non-coding RNAs, especially long non-coding RNAs (lncRNAs) have been reported of contribution to complex disorders like cancer and cardiovascular diseases, for example, the lncRNA MIAT promotes medulloblastoma tumorigenesis and treatment resistance [8]. Compared to coding genes, knowledge for phenotypic effects and consequences of ncRNA variants are largely missing. To assess the effects of ncRNA variants quantitatively between the two phenotypes, we counted the number of exonic variants in ncRNAs, respectively (Fig. 1F). A total of 286 overlapped ncRNAs have at least one exonic variant, and 51 out of 286 ncRNAs (17.8%) have exonic variants in both BD-cancer and BD-only groups. The majority of exonic ERVars in ncRNAs occur in different ncRNAs. LINC01140 is a regulator in osteosarcoma proliferation and invasion [9], with five different variants occurred only in BD-cancer children. There are 3 variants in the adjacent regions: chr1:87132127-G-GA, chr1:87132128-T-TA and chr1:87132128-T- TTTTAATGATA, and the other 2 variants are at the same chromosomal position chr1:87168368-G-A/T. These results indicate the exonic variants may affect different ncRNAs.

ERVars in intronic regions, especially variants close to exons, may disrupt RNA splicing, resulting in the loss of exons or the inclusion of introns, and an altered protein sequence [10]. Intronic variants have attracted significant attention in recent years and have been shown of contribution to tumorigenesis by acting on splicing codes [11]. The average distances were calculated for the 5,708 overlapped genes with intronic variants, and the genes with the closest intronic ERVars are significantly different between the two clinical phenotypes. As shown in Fig. 1G H, the genes with intronic variant distance < 500 bp, have hardly any overlaps. These results suggest that, although a large portion of the genes are overlapped in terms of intronic ERVars, about one-half of the genes are more likely to be altered in BD-cancer patients because the intronic variants are much closer to exon compared to their distances in the BD-only patients. As the threshold increases, the distance differences between BD-cancer and BD-only groups are reduced, indicating the impacts of intronic variants are fading and cause less differences when they are further from the exons. Several interesting genes were uncovered, including *CHD2, PAX3, PAX6*, and *GRHL3*. The intronic variant at chr15: 92,937,746 T->C in *CHD2*, is 197 bp away from exon 5/6 in BD-cancer patients, compared to 1,384 bp in BD-only patients. This variant has the potential to result in alternative splicing, leading to a different transcript ENST00000628375.2 in the BD-cancer patients. For the genes containing intronic ERVars exclusively seen in BD-cancer patients, we observed an enrichment in the coenzyme metabolic process (FDR = 0.014), nicotinate and nicotinamide metabolism (FDR = 0.026), and pentose and glucuronate interconversions pathway (FDR = 0.036). Coenzymes play important roles in cancer metabolism, including pediatric brain tumors such as glioblastoma and neuroblastoma [12]. The enzymes in nicotinate and nicotinamide metabolism, such as nicotinate phosphoribosyl-transferase (NAPRT) may be involved in multiple adult and pediatric tumors [13]. Pentose and glucuronate interconversion is a metabolic pathway altered across all tumor types [14].

## Unique ERVars in 5’ UTR regions

Another notable finding in this study is that genes containing ERVars in 5’UTR regions are most mutually exclusive for the two distinguished clinical phenotypes. In 5’UTR regions, variants may affect upstream open reading frame (uORF) to regulate translation initiation of specific transcripts, and a single-point mutation may create a novel uORF in the tumor suppressor [15]. The functional analysis for BD-cancer specific genes (Supplementary Table 3) showed enrichment of genes in positive regulation of protein serine/threonine kinase activity (FDR = 0.035). Eight genes, including *MSH6, PIK3R1, WRN, PTCH1, NUP98, CLTC*, with variants in 5’ UTR regions, were replicated in the pediatric cancer gene set (Table 1). Overall, the results indicate that variants in 5’UTR regions may contribute for cancer development in BD children by impacting tumorigenesis through the abnormal translation of genes that are critical for protein serine/threonine kinase activities.

**Table 1:** Corresponding genes mapped from exclusive recurrent 5’ UTR variants in BD-cancer patients that overlapped with well-known pediatric genes

Chr	Locus	Ref	Alt	Gene	Alternations	dbSNP
2	47,783,971	G	C	MSH6	NM_001281494:c.-14919G > C	rs531348092
5	68,288,498	C	CCCA	PIK3R1	NM_181524:c.-238 C > CCCA	rs763393624
5	68,288,499	G	C	PIK3R1	NM_181524:c.-237G > C	.
8	31,058,397	T	C	WRN	NM_000553:c.-51T > C	rs187869929
9	95,507,024	A	C	PTCH1	NM_001083607:c.-21209T > G	rs189960172
11	3,797,572	G	A	NUP98	NM_139132:c.-15,455 C > T;NM_016320:c.-15,455 C > T;NM_005387:c.-15,455 C > T;NM_139131:c.-15,455 C > T	rs530477112
17	59,619,872	G	A	CLTC	NM_001288653:c.-260G > A;NM_004859:c.-260G > A	rs140454216
17	59,620,041	C	A	CLTC	NM_001288653:c.-91 C > A;NM_004859:c.-91 C > A	rs185248699

## Conclusion

This first genomic study exploring the impact of recurrent variants exclusive to the two distinguished clinical phenotypes, BD with or without cancer, demonstrates enrichment of selective protein-coding/non-coding RNAsthat are differentially expressed between these two phenotypes, suggesting that selective genetic factors may underlie the molecular processes of pediatric cancer development in BD children. Further study to compare ERVars identified in cancer patients (without BD) with those in BD-cancer patients will gain significant knowledge.

### Electronic supplementary material

Below is the link to the electronic supplementary material.

**Supplementary Material 1**: Supplementary Fig. 1. Venn diagrams for overlapping protein-coding/non-coding RNAs. [a] exonic ERVars; [b] intronic ERVars; [c] non-coding RNA ERVars; [d] ERVars in 5’ UTR regions; [e] ERVars in 3’ UTR regions

**Supplementary Material 2**: Supplementary Table 1. Annotations for exclusive recurrent coding variants in [a] BD-cancer patients; [b] BD-only patients

**Supplementary Material 3**: Supplementary Table 2. Corresponding genes from selected exclusive recurrent variants that overlapped with well-known pediatric cancer genes: [a] genes containing exclusive recurrent coding variants; [b] genes containing exclusive recurrent intronic variants; [c] genes containing exclusive recurrent 3’ UTR variants

**Supplementary Material 4**: Supplementary Table 3. Exclusive recurrent 5’UTR variants in BD-cancer patients

**Supplementary Material 5**: Supplementary Methods

## Data Availability

The KidFirst data could be accessed at Kids First Data Resource Portal (DRC) (https://portal.kidsfirstdrc.org/login).

## List of abbreviations

BD: Birth defects
CAG: Center for Applied Genomics
CHOP: The Children’s Hospital of Philadelphia
DRAGEN: Dynamic Read Analysis for Genomics Bio-IT
EMRs: Electronic medical records
ERVar: Exclusive recurrent variants
ICD code: International Classification of Diseases code
VCF: Variant call format
WGS: Whole genome sequencing

## References

[1] Lupo PJ, Schraw JM, Desrosiers TA, Nembhard WN, Langlois PH, Canfield MA, Copeland G, Meyer RE, Brown AL, Chambers TM (2019). Association between Birth Defects and Cancer Risk among Children and Adolescents in a Population-Based Assessment of 10 million live births. JAMA Oncol.

[2] Liu Y, Qu HQ, Chang X, Mentch FD, Qiu H, Nguyen K, Wang X, Saeidian AH, Watson D, Glessner J, Hakonarson H (2022). Identification of risk variants related to malignant tumors in children with birth defects by whole genome sequencing. Biomark Res.

[3] Vogelstein B, Kinzler KW (2004). Cancer genes and the pathways they control. Nat Med.

[4] Sharma Y, Miladi M, Dukare S, Boulay K, Caudron-Herger M, Gross M, Backofen R, Diederichs S (2019). A pan-cancer analysis of synonymous mutations. Nat Commun.

[5] Zhao F, Wang X, Wang Y, Zhang J, Lai R, Zhang B, Zhou X (2020). The function of uterine UDP-glucuronosyltransferase 1A8 (UGT1A8) and UDP-glucuronosyltransferase 2B7 (UGT2B7) is involved in endometrial cancer based on estrogen metabolism regulation. Horm (Athens).

[6] Falkowski S, Woillard JB, Postil D, Tubiana-Mathieu N, Terrebonne E, Pariente A, Smith D, Guimbaud R, Thalamas C, Rouguieg-Malki K (2017). Common variants in glucuronidation enzymes and membrane transporters as potential risk factors for colorectal cancer: a case control study. BMC Cancer.

[7] Zhang J, Walsh MF, Wu G, Edmonson MN, Gruber TA, Easton J, Hedges D, Ma X, Zhou X, Yergeau DA (2015). Germline mutations in predisposition genes in Pediatric Cancer. N Engl J Med.

[8] Peng KL, Vasudevan HN, Lockney DT, Baum R, Hendrickson RC, Raleigh DR, Schmitt AM (2022). Miat and interacting protein metadherin maintain a stem-like niche to promote medulloblastoma tumorigenesis and treatment resistance. Proc Natl Acad Sci U S A.

[9] Zhang S, Chen R (2022). LINC01140 regulates osteosarcoma proliferation and invasion by targeting the miR-139-5p/HOXA9 axis. Biochem Biophys Rep.

[10] Baralle D, Baralle M (2005). Splicing in action: assessing disease causing sequence changes. J Med Genet.

[11] Jung H, Lee KS, Choi JK (2021). Comprehensive characterisation of intronic mis-splicing mutations in human cancers. Oncogene.

[12] Thapa M, Dallmann G (2020). Role of coenzymes in cancer metabolism. Semin Cell Dev Biol.

[13] Navas LE, Carnero A (2021). NAD(+) metabolism, stemness, the immune response, and cancer. Signal Transduct Target Ther.

[14] Rosario SR, Long MD, Affronti HC, Rowsam AM, Eng KH, Smiraglia DJ (2018). Pan-cancer analysis of transcriptional metabolic dysregulation using the Cancer Genome Atlas. Nat Commun.

[15] Schuster SL, Hsieh AC (2019). The untranslated regions of mRNAs in Cancer. Trends Cancer.

