# Supplementary figures and images for: Genomic variants exclusively identified in children with birth defects and concurrent malignant tumors predispose to cancer development
Source: Mol Cancer. 2023 Aug 5;22:126. doi: 10.1186/s12943-023-01828-5 (PMC10403830; doi:10.1186/s12943-023-01828-5)

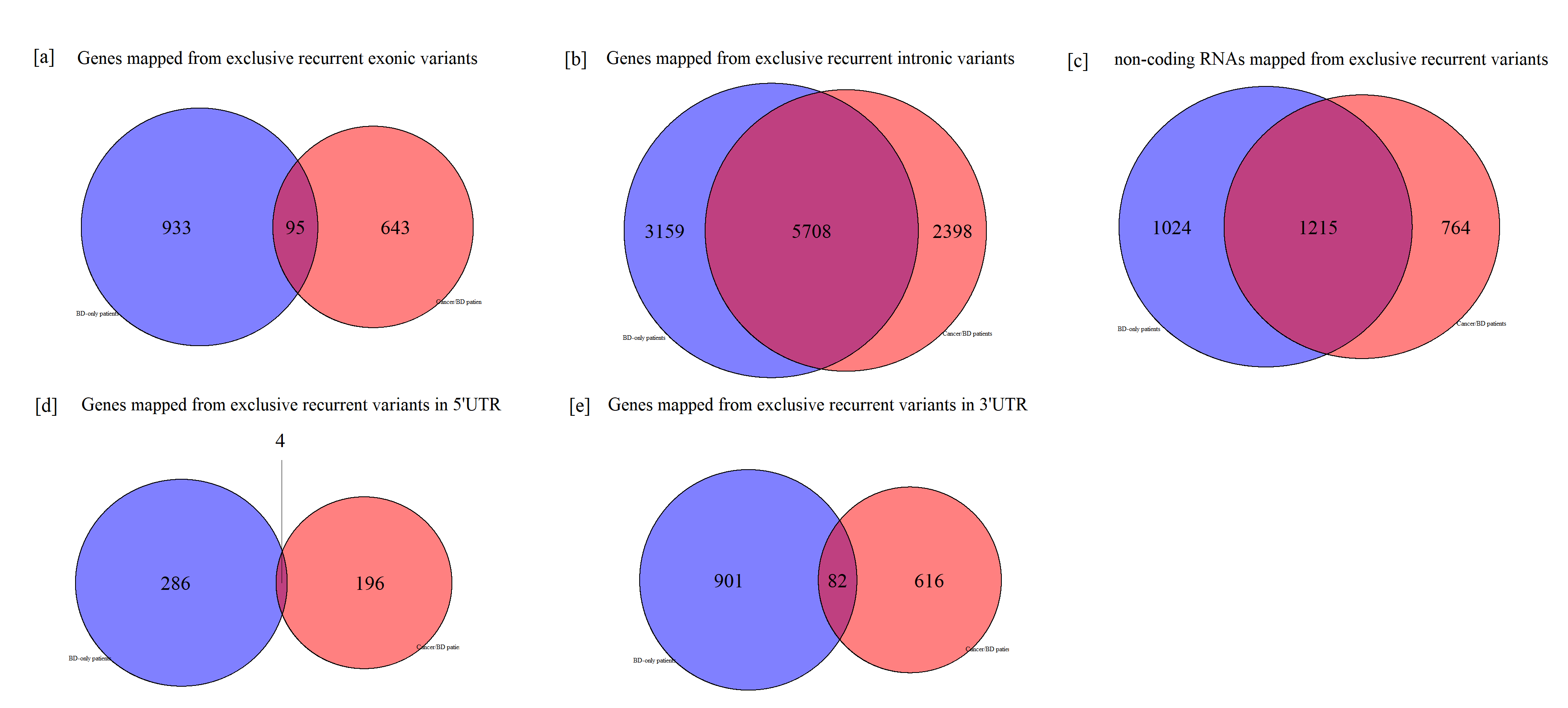

Supplement: Supplementary file 1 — Supplementary Material 1: Supplementary Fig. 1. Venn diagrams for overlapping protein-coding/non-coding RNAs. [a] exonic ERVars; [b] intronic ERVars; [c] non-coding RNA ERVars; [d] ERVars in 5’ UTR regions; [e] ERVars in 3’ UTR regions [file 12943_2023_1828_MOESM1_ESM.png]
