# Supplementary material for: Genomic variants exclusively identified in children with birth defects and concurrent malignant tumors predispose to cancer development
Source: Mol Cancer. 2023 Aug 5;22:126. doi: 10.1186/s12943-023-01828-5 (PMC10403830; doi:10.1186/s12943-023-01828-5)
Supplement: Supplementary file 5 — Supplementary Material 5: Supplementary Methods [file 12943_2023_1828_MOESM5_ESM.docx]

**Genomic Variants Exclusively Identified in Children with Birth Defects and Concurrent Malignant Tumors Predispose to Cancer Development**

**Supplementary Methods**

**Patient recruitment**

The patients with birth defects (BD), with or without co-occurrence of pediatric onset cancers, were recruited by the Center for Applied Genomics (CAG), the Children’s Hospital of Philadelphia (CHOP). The BD and cancer diagnosis used the International Classification of Diseases (ICD) codes ICD-9/ ICD-10. All the CAG patients were recorded in the electronic medical records (EMRs) of CHOP established in 2003. CAG at CHOP maintains a de-identified extract of clinical data from the CHOP EMR and EHR databases of consented patients. This database contains longitudinal information about visits, diagnoses, medical history, prescriptions, procedures, and lab tests with all information coded and de-identified.

Altogether, 1221 probands with non-chromosomal anomalies were studied, including 454 BD patients with at least one type of malignant tumors (BD-cancer) and 767 BD patients without any known cancers (BD-only). In addition, 345 healthy controls without BD or cancer who were parents/siblings of the probands were also investigated in comparison. All the patients were recruited during regular hospital visits at multiple clinical settings, including emergency room, ambulatory settings or surgical settings, through the general pediatric clinics or CHOP’s pediatric specialty practices. The patients were in the age range of 0-21 years and receiving health care at CHOP. Parental consent was obtained for individuals under 18 years of age and assent was also obtained for subjects aged 7-17 years. The informed consent allows samples to be obtained and analyzed using the genomic technologies in this study, to address the proposed research questions.

**Processing and variant detection by WGS**

Whole genome sequencing (WGS) was done at 30X coverage for the 1566 individuals as a part of the Gabriella Miller Kids First project, by the genomics platform of the Broad Institute (MIT) using the same protocol (HISeq X 10) to avoid any potential technical/batch biases. The variant call format (VCF) files of WGS were generated using the Illumina DRAGEN (Dynamic Read Analysis for GENomics) Bio-IT Platform (Illumina, San Diego, CA), aligned to the GRCh38/hg38 human genome assembly. Variants were only retained for those with “PASS” in the quality control field, and the sequencing depth (DP) greater than 30. The annotations for the variants were generated using the ANNOVAR software developed by our group with default parameters [1], and the variants were further classified into variants in coding regions, introns, 5’ or 3’ untranslated regions (UTR), and non-coding RNA regions.

**ERVars in BD-cancer versus BD-only patients**

The ancestral origins of all the subjects have been confirmed by the PCA analysis of genome-wide common variants. The ethnicity distributions of BD-cancer and BD-only are mainly Caucasian in each group (75% vs 70% without statistical significance, *P*=0.0604). A variant is considered “identical” if the mutation is at the same genomic locus with the same alternative allele. Variants that occur in at least three individuals in the 454 BD patients with malignant tumors and were absent in the 767 BD-only patients and the 345 healthy family controls, were identified as recurrent malignant cancer specific variants. Similarly, variants that occur in at least three individuals in the 767 BD cancer-free patients and were absent in the 454 BD patients with malignant tumors and the 345 healthy family controls, were identified as recurrent BD specific variants. For ERVars identified in BD-cancer, the variants identified in the patients from more than one ethnicity were 51%. The results were very unlikely to be due to the frequencies of the variants in the populations, as the two groups were selected from the same ethnic backgrounds, and more than half of the variants were not population specific. Selected variants were then mapped to the corresponding genes/non-coding RNAs based on their genomic location on GRCh38/hg38 human genome assembly. If ERVars in BD-cancer and BD-only patients mapped to the same gene/non-coding RNA, these are defined as the “overlapped”.

**Function of Overlapped Genes Impacted by ERVars**

For variants in coding regions, the exon number and strand direction were identified based on the GRCh38/hg38 GENCODE human genome reference. If multiple variants were mapped to the same gene, the average exon numbers was calculated for overlapped variants. Then, the target exon numbers were then normalized by the total exon count of the corresponding gene. For variants mapped into intronic regions, the distances in base pair (bp) to the closest exon were calculated based on the GRCh38/hg38 GENCODE human genome reference. If there were multiple intronic variants identified in the same gene, average distance was obtained. For non-coding RNAs (ncRNA), number of variants in ncRNA exonic regions and intronic regions were counted separately for overlap. Functional enrichment analysis was performed using the DAVID bioinformatics platform [2] and the WebGestalt (WEB-based Gene SeT AnaLysis Toolkit) [3].

**References**

1. Wang K, Li M, Hakonarson H: **ANNOVAR: functional annotation of genetic variants from high-throughput sequencing data.** *Nucleic Acids Res* 2010, **38:**e164.

2. Sherman BT, Hao M, Qiu J, Jiao X, Baseler MW, Lane HC, Imamichi T, Chang W: **DAVID: a web server for functional enrichment analysis and functional annotation of gene lists (2021 update).** *Nucleic Acids Res* 2022, **50:**W216-221.

3. Liao Y, Wang J, Jaehnig EJ, Shi Z, Zhang B: **WebGestalt 2019: gene set analysis toolkit with revamped UIs and APIs.** *Nucleic Acids Res* 2019, **47:**W199-W205.
